# Supplementary material for: Humanized Klotho haplotypes cause widespread transcriptomic changes in mouse brain
Source: bioRxiv. 2026 Apr 16:2026.04.15.718745. Preprint. [Version 1] doi: 10.64898/2026.04.15.718745 (PMC13105077; doi:10.64898/2026.04.15.718745)
Supplement: 1 [file NIHPP2026.04.15.718745V1-supplement-1.pdf]

bioRxiv preprint doi: https://doi.org/10.64898/2026.04.15.718745; this version posted April 16, 2026. The copyright holder for this preprint (which was not certified by peer review) is the author/funder, who has granted bioRxiv a license to display the preprint in perpetuity. It is made available under aCC-BY 4.0 International license.

505 # Supplemental Figures

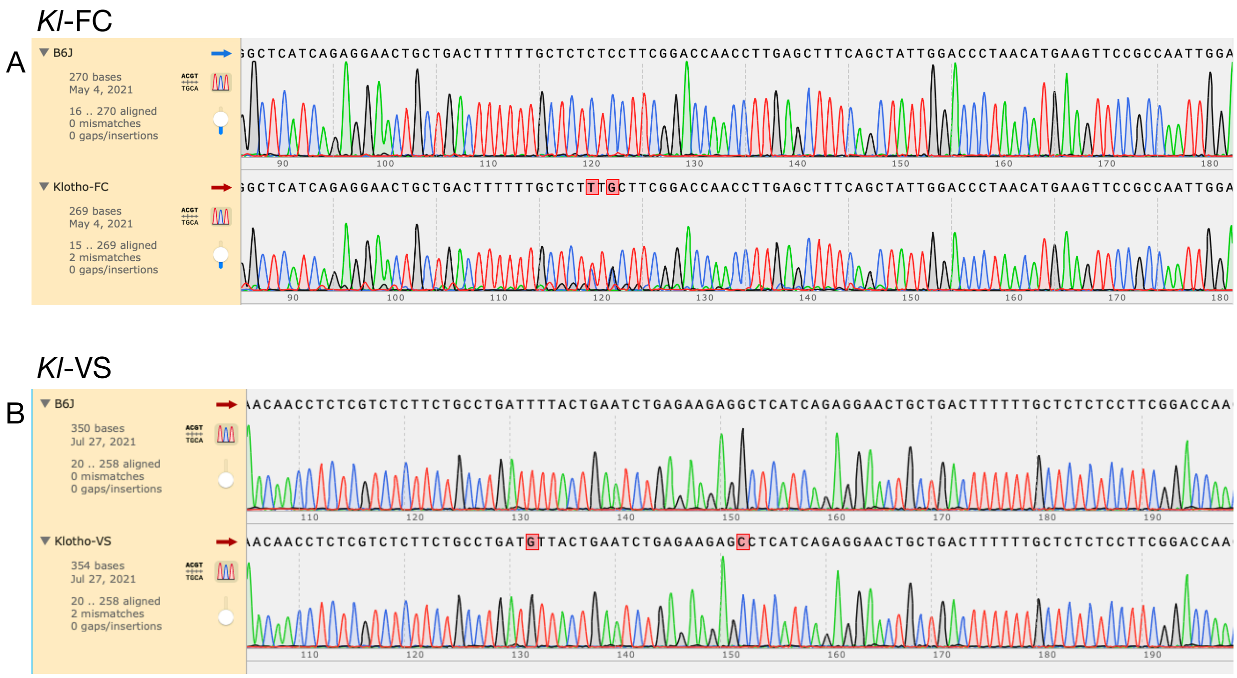

Figure 1: Sequencing results for humanized *Kl* alleles compared to C57BL/6J (B6) reference. **(A)** Sequencing results for the *Kl*-FC construct (bottom) compared to the B6 reference (top). Highlighted bases show the SNP used to introduce the S370C variant (TCC>TGC), as well as a silent mutation (CTC>CTT) immediately downstream used to prevent re-cutting. **(B)** Sequencing results for the *Kl*-VS construct (bottom) compared to the B6 reference (top). Highlighted bases indicate the SNP used to introduce the F352V variant (TTT>GTT) as well as a silent mutation (AGG>AGC) immediately downstream used to prevent re-cutting.

bioRxiv preprint doi: https://doi.org/10.64898/2026.04.15.718745; this version posted April 16, 2026. The copyright holder for this preprint (which was not certified by peer review) is the author/funder, who has granted bioRxiv a license to display the preprint in perpetuity. It is made available under aCC-BY 4.0 International license.

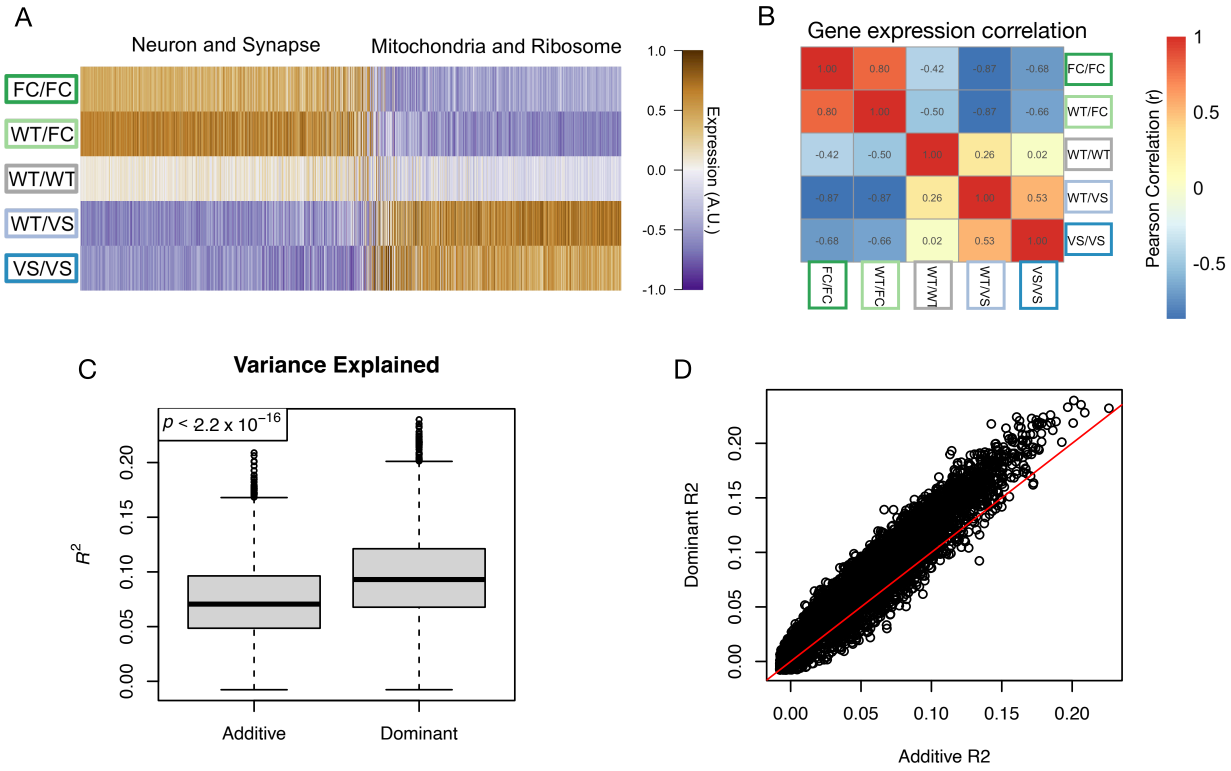

Figure 2: Justification for grouping allele carriers together. **A.** Mean expression for all genes significantly differentially expressed across the genotypes. Values shown are the mean expression for each genotype with genes shown in columns and genotypes shown in rows. The expression clusters into two major clusters enriched for neuronal processes and mitochondrial processes as indicated. The FC carriers tend to have correlated gene expression across the clusters, as do the VS carriers. **B.** Gene expression correlations between genotype pairs. The FC carriers are correlated with each other and the VS carriers are correlated with each other. FC and VS carriers have anti-correlated gene expression. **C.** Comparison of variance explained for additive and dominant linear models for all significantly differentially expressed genes. The dominant coding explained significnatly more variance than the additive coding. **D.** Comparison of statistics from linear models using dominant and additive codings. The dominant coding tended to have higher variance explained than the additive coding across the range of variance explained, but particularly in genes with high variance explained by genotype. Red line shows $y = x$.

bioRxiv preprint doi: https://doi.org/10.64898/2026.04.15.718745; this version posted April 16, 2026. The copyright holder for this preprint (which was not certified by peer review) is the author/funder, who has granted bioRxiv a license to display the preprint in perpetuity. It is made available under aCC-BY 4.0 International license.

Functional enrichments of differentially expressed gene clusters

Cluster 1

term	N−term	N−query	overlap	p value	domain
mitochondrion	1900	1800	420	5.9e−109	GO:CC
ribosomal subunit	200	1800	140	1.1e−108	GO:CC
mitochondrial envelope	840	1800	260	2.3e−99	GO:CC
mitochondrial membrane	780	1800	240	3.4e−92	GO:CC
mitochondrial inner membrane	530	1800	200	1e−91	GO:CC
organelle envelope	1300	1800	310	2.1e−91	GO:CC
organelle inner membrane	580	1800	200	1.3e−85	GO:CC
translation	720	1800	210	3.9e−76	GO:BP
cytosolic ribosome	130	1800	92	1.8e−75	GO:CC
cytoplasmic translation	160	1800	99	1.7e−70	GO:BP
parkinson disease	260	950	140	4.8e−67	KEGG
ribosome	430	1800	150	6.3e−64	GO:CC
large ribosomal subunit	130	1800	81	4.1e−60	GO:CC
aerobic respiration	200	1800	100	1.1e−59	GO:BP
huntington disease	300	950	140	2.3e−59	KEGG

Cluster 2

term	N−term	N−query	overlap	p value	domain
nervous system development	2500	1700	430	1.3e−83	GO:BP
cell junction	2300	1700	370	1.9e−67	GO:CC
neuron development	1300	1700	270	1.2e−65	GO:BP
generation of neurons	1600	1700	310	1.4e−65	GO:BP
neuron projection development	1100	1700	250	2.1e−64	GO:BP
neuron projection	1500	1700	290	3.5e−64	GO:CC
synapse	1600	1700	300	4.9e−64	GO:CC
neurogenesis	1900	1700	330	2e−63	GO:BP
neuron differentiation	1600	1700	290	4e−62	GO:BP
cell projection	2600	1700	390	2e−58	GO:CC
plasma membrane bounded cell projection ...	1700	1700	290	1.1e−53	GO:BP
cell projection organization	1700	1700	290	1.9e−52	GO:BP
axon	800	1700	180	2.1e−51	GO:CC
somatodendritic compartment	1100	1700	220	7.1e−51	GO:CC
plasma membrane bounded cell projection	2500	1700	360	7.5e−51	GO:CC

Figure 3: Functional enrichments of genes shown in Fig. 2B. Genes in cluster 1 were relatively up-regulated in VS carriers and were enriched in mitochondrial and ribosomal functions. The top 15 most enriched terms are shown here. Genes in cluster 2 were relatively down-regulated in VS carriers and were enriched in neuronal and synaptic functions. The top 15 most enriched terms are shown here.

Enrichment of genes with differential exon usage

term	N-term	N-query	overlap	p value	domain
synapse	1900	1800	350	2.8e-70	GO:CC
cell junction	2500	1800	410	2.9e-68	GO:CC
postsynapse	1000	1800	220	1.2e-57	GO:CC
regulation of cellular component organization	2500	1800	380	7.5e-51	GO:BP
glutamatergic synapse	810	1800	190	1.1e-50	GO:CC
cell projection	2600	1800	380	2.6e-50	GO:CC
neuron projection	1500	1800	270	2.9e-48	GO:CC
plasma membrane bounded cell projection	2500	1800	360	1.4e-45	GO:CC
enzyme binding	2200	1700	350	5e-45	GO:MF
cellular macromolecule localization	2600	1800	370	3.1e-43	GO:BP
nervous system development	2600	1800	370	3.2e-43	GO:BP
somatodendritic compartment	1100	1800	210	4.9e-43	GO:CC
protein localization	2600	1800	370	7.8e-43	GO:BP
establishment of localization in cell	2000	1800	310	3.4e-41	GO:BP
purine ribonucleotide binding	2100	1700	320	2.4e-36	GO:MF
neuron projection development	1100	1800	200	7.4e-36	GO:BP
vesicle-mediated transport	1500	1800	250	7.6e-36	GO:BP
purine ribonucleoside triphosphate binding	2100	1700	310	1.1e-35	GO:MF
neuron development	1300	1800	220	1.2e-35	GO:BP
ribonucleotide binding	2200	1700	320	1.5e-35	GO:MF
plasma membrane bounded cell projection organization	1700	1800	260	2.7e-35	GO:BP
neuron to neuron synapse	500	1800	120	9.5e-35	GO:CC
dendrite	800	1800	160	1.9e-34	GO:CC
vesicle	2200	1800	310	2.2e-34	GO:CC
dendritic tree	800	1800	160	2.6e-34	GO:CC
neurogenesis	1900	1800	280	5.6e-34	GO:BP
generation of neurons	1700	1800	260	6.5e-34	GO:BP
intracellular vesicle	2100	1800	290	6.5e-34	GO:CC
purine nucleotide binding	2200	1700	330	7.6e-34	GO:MF
anion binding	2700	1700	370	1.3e-33	GO:MF

Figure 4: Functional enrichments of genes with significantly varying exon usage across *Kl* haplotypes.

bioRxiv preprint doi: https://doi.org/10.64898/2026.04.15.718745; this version posted April 16, 2026. The copyright holder for this preprint (which was not certified by peer review) is the author/funder, who has granted bioRxiv a license to display the preprint in perpetuity. It is made available under aCC-BY 4.0 International license.

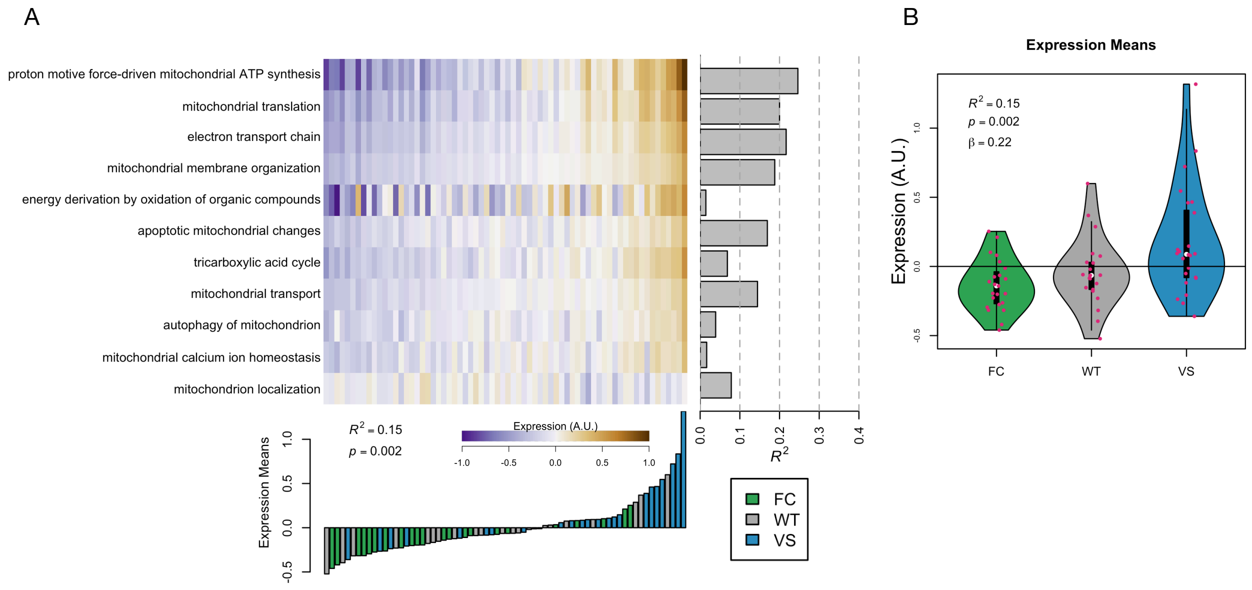

Figure 5: Mitochondrial metabolism subdomains. **A** (Above) Average expression of each subdomain in the Biodomain Mitochondrial Metabolism. Negative values are shown in purple, and positive values are shown in brown. Gray bars indicate the variance explained in expression by genotype. (Below) Average expression across all subdomains for each individual mouse. The color of each bar indicates the mouse's *Kl* haplotype. **B** Violin plot showing the overall expression of the Biodomain separated by genotype.

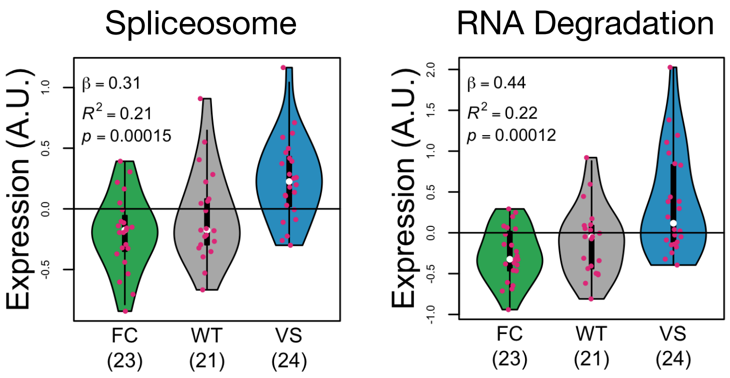

Figure 6: Intersections of the Biodomain RNA Spliceosome and KEGG pathways were differentially expressed, but the adjusted p values were slightly above the cutoff used in the intersectional analysis. Titles on each panel indicate the name of the KEGG pathway that was intersected with the RNA Spliceosome Biodomain.
